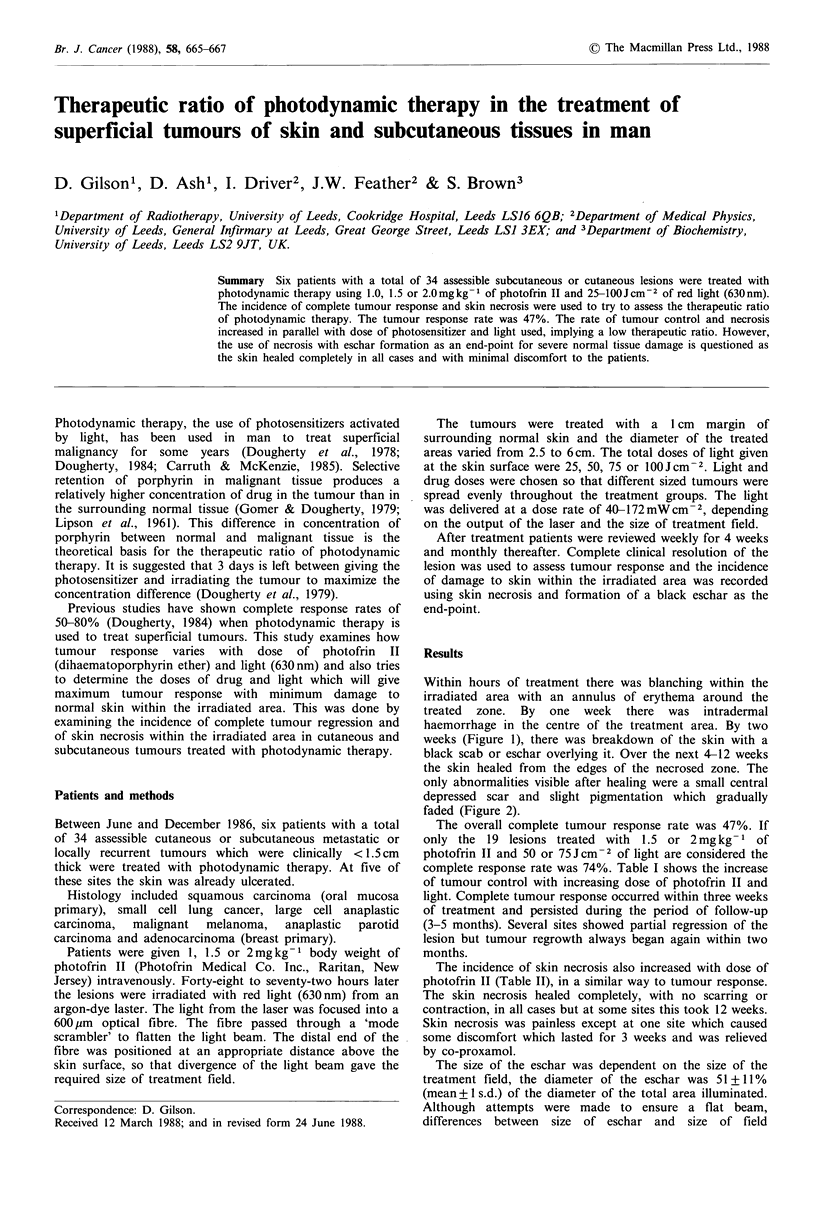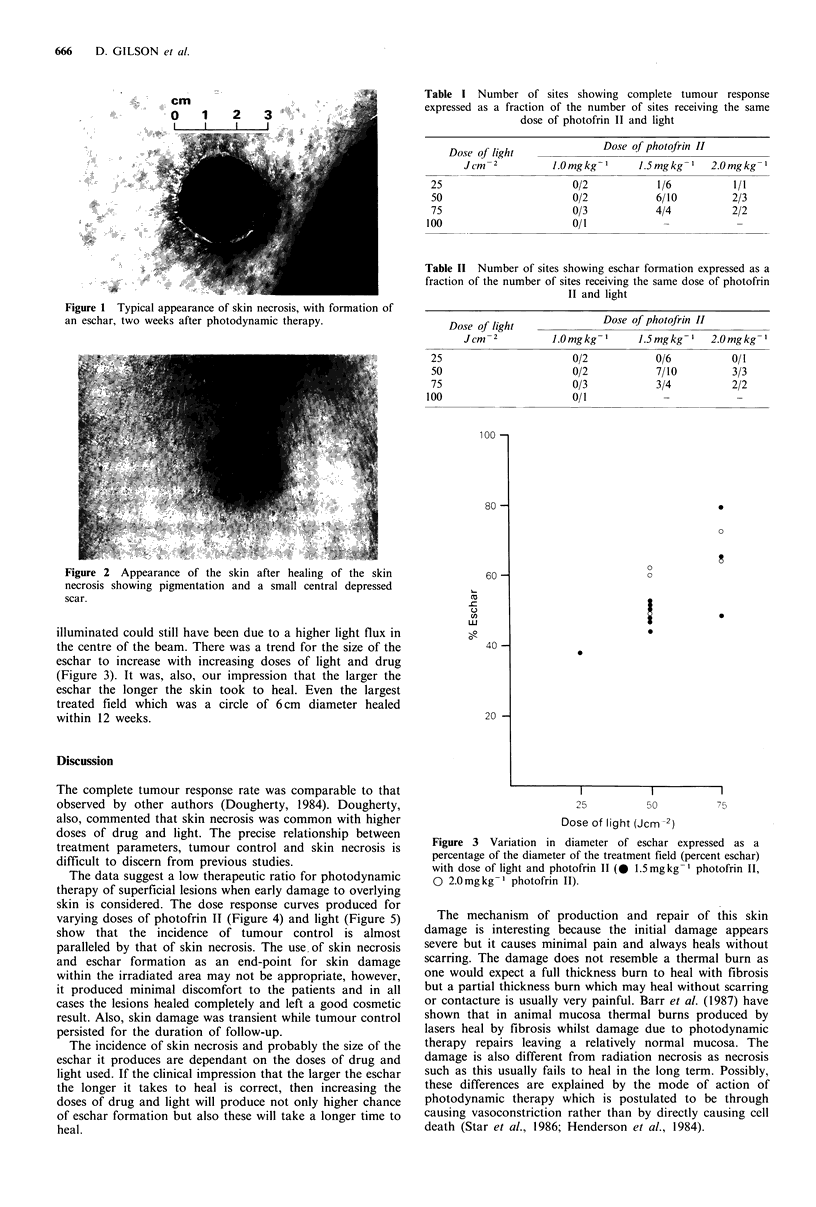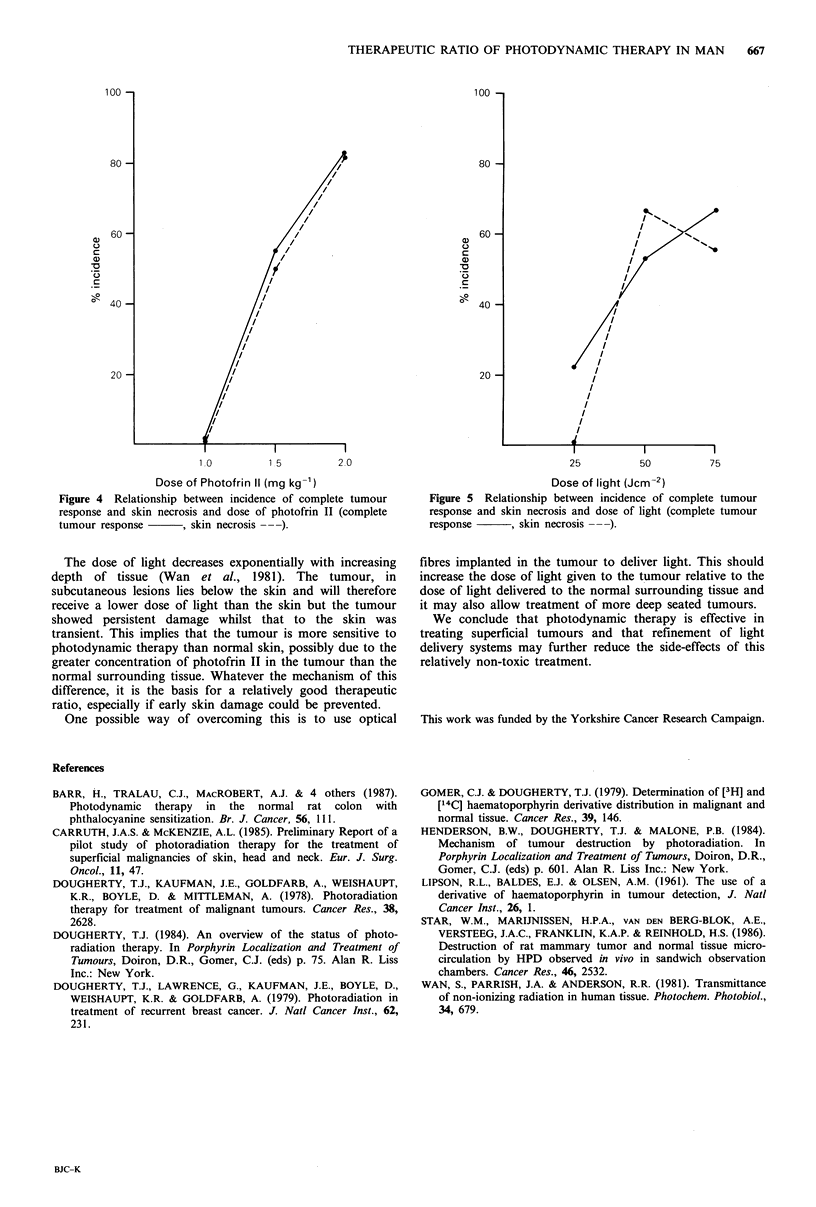# Therapeutic ratio of photodynamic therapy in the treatment of superficial tumours of skin and subcutaneous tissues in man.

**DOI:** 10.1038/bjc.1988.282

**Published:** 1988-11

**Authors:** D. Gilson, D. Ash, I. Driver, J. W. Feather, S. Brown

**Affiliations:** Department of Radiotherapy, University of Leeds, Cookridge Hospital, Leeds, UK.

## Abstract

**Images:**


© The Macmillan Press Ltd., 1988

### Therapeutic ratio of photodynamic therapy in the treatment of superficial tumours of skin and subcutaneous tissues in man

D. Gilson[1], D. Ash[1], I. Driver[2], J.W. Feather[2] & S. Brown[3]

[1]*Department of Radiotherapy, University of Leeds, Cookridge Hospital, Leeds LS16 6QB;* [2]*Department of Medical Physics, University of Leeds, General Infirmary at Leeds, Great George Street, Leeds LS1 3EX; and* [3]*Department of Biochemistry, University of Leeds, Leeds LS2 9JT, UK.*

**Summary** Six patients with a total of 34 assessible subcutaneous or cutaneous lesions were treated with photodynamic therapy using 1.0, 1.5 or 2.0 $mg\,kg^{-1}$ of photofrin II and 25–100 $J\,cm^{-2}$ of red light (630 nm). The incidence of complete tumour response and skin necrosis were used to try to assess the therapeutic ratio of photodynamic therapy. The tumour response rate was 47%. The rate of tumour control and necrosis increased in parallel with dose of photosensitizer and light used, implying a low therapeutic ratio. However, the use of necrosis with eschar formation as an end-point for severe normal tissue damage is questioned as the skin healed completely in all cases and with minimal discomfort to the patients.

Photodynamic therapy, the use of photosensitizers activated by light, has been used in man to treat superficial malignancy for some years (Dougherty *et al.*, 1978; Dougherty, 1984; Carruth & McKenzie, 1985). Selective retention of porphyrin in malignant tissue produces a relatively higher concentration of drug in the tumour than in the surrounding normal tissue (Gomer & Dougherty, 1979; Lipson *et al.*, 1961). This difference in concentration of porphyrin between normal and malignant tissue is the theoretical basis for the therapeutic ratio of photodynamic therapy. It is suggested that 3 days is left between giving the photosensitizer and irradiating the tumour to maximize the concentration difference (Dougherty *et al.*, 1979).

Previous studies have shown complete response rates of 50–80% (Dougherty, 1984) when photodynamic therapy is used to treat superficial tumours. This study examines how tumour response varies with dose of photofrin II (dihaematoporphyrin ether) and light (630 nm) and also tries to determine the doses of drug and light which will give maximum tumour response with minimum damage to normal skin within the irradiated area. This was done by examining the incidence of complete tumour regression and of skin necrosis within the irradiated area in cutaneous and subcutaneous tumours treated with photodynamic therapy.

### Patients and methods

Between June and December 1986, six patients with a total of 34 assessible cutaneous or subcutaneous metastatic or locally recurrent tumours which were clinically $<1.5$ cm thick were treated with photodynamic therapy. At five of these sites the skin was already ulcerated.

Histology included squamous carcinoma (oral mucosa primary), small cell lung cancer, large cell anaplastic carcinoma, malignant melanoma, anaplastic parotid carcinoma and adenocarcinoma (breast primary).

Patients were given 1, 1.5 or 2 $mg\,kg^{-1}$ body weight of photofrin II (Photofrin Medical Co. Inc., Raritan, New Jersey) intravenously. Forty-eight to seventy-two hours later the lesions were irradiated with red light (630 nm) from an argon-dye laster. The light from the laser was focused into a 600 $\mu m$ optical fibre. The fibre passed through a 'mode scrambler' to flatten the light beam. The distal end of the fibre was positioned at an appropriate distance above the skin surface, so that divergence of the light beam gave the required size of treatment field.

Correspondence: D. Gilson.
Received 12 March 1988; and in revised form 24 June 1988.

The tumours were treated with a 1 cm margin of surrounding normal skin and the diameter of the treated areas varied from 2.5 to 6 cm. The total doses of light given at the skin surface were 25, 50, 75 or 100 $J\,cm^{-2}$. Light and drug doses were chosen so that different sized tumours were spread evenly throughout the treatment groups. The light was delivered at a dose rate of 40–172 $mW\,cm^{-2}$, depending on the output of the laser and the size of treatment field.

After treatment patients were reviewed weekly for 4 weeks and monthly thereafter. Complete clinical resolution of the lesion was used to assess tumour response and the incidence of damage to skin within the irradiated area was recorded using skin necrosis and formation of a black eschar as the end-point.

### Results

Within hours of treatment there was blanching within the irradiated area with an annulus of erythema around the treated zone. By one week there was intradermal haemorrhage in the centre of the treatment area. By two weeks (Figure 1), there was breakdown of the skin with a black scab or eschar overlying it. Over the next 4–12 weeks the skin healed from the edges of the necrosed zone. The only abnormalities visible after healing were a small central depressed scar and slight pigmentation which gradually faded (Figure 2).

The overall complete tumour response rate was 47%. If only the 19 lesions treated with 1.5 or 2 $mg\,kg^{-1}$ of photofrin II and 50 or 75 $J\,cm^{-2}$ of light are considered the complete response rate was 74%. Table I shows the increase of tumour control with increasing dose of photofrin II and light. Complete tumour response occurred within three weeks of treatment and persisted during the period of follow-up (3–5 months). Several sites showed partial regression of the lesion but tumour regrowth always began again within two months.

The incidence of skin necrosis also increased with dose of photofrin II (Table II), in a similar way to tumour response. The skin necrosis healed completely, with no scarring or contraction, in all cases but at some sites this took 12 weeks. Skin necrosis was painless except at one site which caused some discomfort which lasted for 3 weeks and was relieved by co-proxamol.

The size of the eschar was dependent on the size of the treatment field, the diameter of the eschar was $51 \pm 11\%$ (mean $\pm 1$ s.d.) of the diameter of the total area illuminated. Although attempts were made to ensure a flat beam, differences between size of eschar and size of field

**Figure 1** Typical appearance of skin necrosis, with formation of an eschar, two weeks after photodynamic therapy.

**Figure 2** Appearance of the skin after healing of the skin necrosis showing pigmentation and a small central depressed scar.

illuminated could still have been due to a higher light flux in the centre of the beam. There was a trend for the size of the eschar to increase with increasing doses of light and drug (Figure 3). It was, also, our impression that the larger the eschar the longer the skin took to heal. Even the largest treated field which was a circle of 6 cm diameter healed within 12 weeks.

### Discussion

The complete tumour response rate was comparable to that observed by other authors (Dougherty, 1984). Dougherty, also, commented that skin necrosis was common with higher doses of drug and light. The precise relationship between treatment parameters, tumour control and skin necrosis is difficult to discern from previous studies.

The data suggest a low therapeutic ratio for photodynamic therapy of superficial lesions when early damage to overlying skin is considered. The dose response curves produced for varying doses of photofrin II (Figure 4) and light (Figure 5) show that the incidence of tumour control is almost paralleled by that of skin necrosis. The use of skin necrosis and eschar formation as an end-point for skin damage within the irradiated area may not be appropriate, however, it produced minimal discomfort to the patients and in all cases the lesions healed completely and left a good cosmetic result. Also, skin damage was transient while tumour control persisted for the duration of follow-up.

The incidence of skin necrosis and probably the size of the eschar it produces are dependant on the doses of drug and light used. If the clinical impression that the larger the eschar the longer it takes to heal is correct, then increasing the doses of drug and light will produce not only higher chance of eschar formation but also these will take a longer time to heal.

**Table I** Number of sites showing complete tumour response expressed as a fraction of the number of sites receiving the same dose of photofrin II and light

| *Dose of light* $J cm^{-2}$ | *Dose of photofrin II* | | |
|---|---|---|---|
| | *1.0 mg kg*$^{-1}$ | *1.5 mg kg*$^{-1}$ | *2.0 mg kg*$^{-1}$ |
| 25 | 0/2 | 1/6 | 1/1 |
| 50 | 0/2 | 6/10 | 2/3 |
| 75 | 0/3 | 4/4 | 2/2 |
| 100 | 0/1 | – | – |

**Table II** Number of sites showing eschar formation expressed as a fraction of the number of sites receiving the same dose of photofrin II and light

| *Dose of light* $J cm^{-2}$ | *Dose of photofrin II* | | |
|---|---|---|---|
| | *1.0 mg kg*$^{-1}$ | *1.5 mg kg*$^{-1}$ | *2.0 mg kg*$^{-1}$ |
| 25 | 0/2 | 0/6 | 0/1 |
| 50 | 0/2 | 7/10 | 3/3 |
| 75 | 0/3 | 3/4 | 2/2 |
| 100 | 0/1 | – | – |

**Figure 3** Variation in diameter of eschar expressed as a percentage of the diameter of the treatment field (percent eschar) with dose of light and photofrin II (● 1.5 mg kg$^{-1}$ photofrin II, ○ 2.0 mg kg$^{-1}$ photofrin II).

The mechanism of production and repair of this skin damage is interesting because the initial damage appears severe but it causes minimal pain and always heals without scarring. The damage does not resemble a thermal burn as one would expect a full thickness burn to heal with fibrosis but a partial thickness burn which may heal without scarring or contacture is usually very painful. Barr *et al.* (1987) have shown that in animal mucosa thermal burns produced by lasers heal by fibrosis whilst damage due to photodynamic therapy repairs leaving a relatively normal mucosa. The damage is also different from radiation necrosis as necrosis such as this usually fails to heal in the long term. Possibly, these differences are explained by the mode of action of photodynamic therapy which is postulated to be through causing vasoconstriction rather than by directly causing cell death (Star *et al.*, 1986; Henderson *et al.*, 1984).

**Figure 4** Relationship between incidence of complete tumour response and skin necrosis and dose of photofrin II (complete tumour response ———, skin necrosis - - -).

**Figure 5** Relationship between incidence of complete tumour response and skin necrosis and dose of light (complete tumour response ———, skin necrosis - - -).

The dose of light decreases exponentially with increasing depth of tissue (Wan *et al.*, 1981). The tumour, in subcutaneous lesions lies below the skin and will therefore receive a lower dose of light than the skin but the tumour showed persistent damage whilst that to the skin was transient. This implies that the tumour is more sensitive to photodynamic therapy than normal skin, possibly due to the greater concentration of photofrin II in the tumour than the normal surrounding tissue. Whatever the mechanism of this difference, it is the basis for a relatively good therapeutic ratio, especially if early skin damage could be prevented.

One possible way of overcoming this is to use optical fibres implanted in the tumour to deliver light. This should increase the dose of light given to the tumour relative to the dose of light delivered to the normal surrounding tissue and it may also allow treatment of more deep seated tumours.

We conclude that photodynamic therapy is effective in treating superficial tumours and that refinement of light delivery systems may further reduce the side-effects of this relatively non-toxic treatment.

This work was funded by the Yorkshire Cancer Research Campaign.

**References**

BARR, H., TRALAU, C.J., MACROBERT, A.J. & 4 others (1987). Photodynamic therapy in the normal rat colon with phthalocyanine sensitization. *Br. J. Cancer,* **56**, 111.

CARRUTH, J.A.S. & MCKENZIE, A.L. (1985). Preliminary Report of a pilot study of photoradiation therapy for the treatment of superficial malignancies of skin, head and neck. *Eur. J. Surg. Oncol.*, **11**, 47.

DOUGHERTY, T.J., KAUFMAN, J.E., GOLDFARB, A., WEISHAUPT, K.R., BOYLE, D. & MITTLEMAN, A. (1978). Photoradiation therapy for treatment of malignant tumours. *Cancer Res.*, **38**, 2628.

DOUGHERTY, T.J. (1984). An overview of the status of photoradiation therapy. In *Porphyrin Localization and Treatment of Tumours*, Doiron, D.R., Gomer, C.J. (eds) p. 75. Alan R. Liss Inc.: New York.

DOUGHERTY, T.J., LAWRENCE, G., KAUFMAN, J.E., BOYLE, D., WEISHAUPT, K.R. & GOLDFARB, A. (1979). Photoradiation in treatment of recurrent breast cancer. *J. Natl Cancer Inst.*, **62**, 231.

GOMER, C.J. & DOUGHERTY, T.J. (1979). Determination of [$^3$H] and [$^{14}$C] haematoporphyrin derivative distribution in malignant and normal tissue. *Cancer Res.*, **39**, 146.

HENDERSON, B.W., DOUGHERTY, T.J. & MALONE, P.B. (1984). Mechanism of tumour destruction by photoradiation. In *Porphyrin Localization and Treatment of Tumours*, Doiron, D.R., Gomer, C.J. (eds) p. 601. Alan R. Liss Inc.: New York.

LIPSON, R.L., BALDES, E.J. & OLSEN, A.M. (1961). The use of a derivative of haematoporphyrin in tumour detection, *J. Natl Cancer Inst.*, **26**, 1.

STAR, W.M., MARIJNISSEN, H.P.A., VAN DEN BERG-BLOK, A.E., VERSTEEG, J.A.C., FRANKLIN, K.A.P. & REINHOLD, H.S. (1986). Destruction of rat mammary tumor and normal tissue microcirculation by HPD observed *in vivo* in sandwich observation chambers. *Cancer Res.*, **46**, 2532.

WAN, S., PARRISH, J.A. & ANDERSON, R.R. (1981). Transmittance of non-ionizing radiation in human tissue. *Photochem. Photobiol.*, **34**, 679.